# *NPY* Gene Methylation in Circulating Tumor DNA as an Early Biomarker for Treatment Effect in Metastatic Colorectal Cancer

**DOI:** 10.3390/cancers14184459

**Published:** 2022-09-14

**Authors:** Louise Raunkilde, Torben Frøstrup Hansen, Rikke Fredslund Andersen, Birgitte Mayland Havelund, Caroline Brenner Thomsen, Lars Henrik Jensen

**Affiliations:** 1 Department of Oncology, Vejle Hospital, University Hospital of Southern, 7100 Vejle, Denmark; 2 Danish Colorectal Cancer Center South, Vejle Hospital, University Hospital of Southern Denmark, 7100 Vejle, Denmark; 3 Department of Regional Health Research, University of Southern Denmark, 5000 Odense, Denmark; 4 Department of Clinical Biochemistry and Immunology, Vejle Hospital, University Hospital of Southern Denmark, 7100 Vejle, Denmark

**Keywords:** circulating tumor DNA, NPY methylation, colorectal cancer, liquid biopsy, droplet digital polymerase chain reaction (ddPCR), monitoring, treatment effect

## Abstract

**Simple Summary:**

Despite several limitations, imaging is still the gold standard in evaluating the treatment effect in metastatic colorectal cancer, and the best response is often achieved several months after treatment start. The present study investigated methylated circulating tumor DNA (ctDNA) in the blood and found that a reduction in ctDNA after one treatment cycle was a better marker of progression-free survival than imaging. Furthermore, ctDNA increases in the blood before progression are visible on imaging, allowing for a change in treatment at an earlier time point. Methylated ctDNA is an early indicator of treatment benefit and a promising endpoint in clinical trials.

**Abstract:**

Despite several limitations, the Response Evaluation Criteria in Solid Tumors version 1.1 (RECIST 1.1) are still the gold standard in response evaluation of metastatic colorectal cancer (mCRC). The aim of the present study was to investigate hypermethylated *neuropeptide Y* circulating tumor DNA (meth-NPY) as an early biomarker for treatment effect and monitoring in 70 mCRC patients receiving first-line treatment in the FOLFOXIRI-Toco trial. Meth-NPY was analyzed using droplet digital PCR, and the response rate was defined as the fraction of patients converting from a baseline detectable level to an undetectable level after the first treatment cycle (responders). A significant increase in meth-NPY was defined as a value with no overlap between the 95% CI of the current and preceding measurement. Progression-free survival (PFS) was significantly longer in meth-NPY responders compared to non-responders, 10.1 and 7.6 months, respectively (*p* = 0.02, HR = 0.43). Patients with response according to RECIST 1.1 had a PFS of 10.1 compared to 7.3 months for non-responders (*p* = 0.17, HR = 0.65). A significant increase in meth-NPY was found with a median of 49 days before radiological progression. In conclusion, early meth-NPY response proved superior to response according to RECIST 1.1 with respect to predicting improved PFS. Meth-NPY is an early indicator of progression, allowing treatment reorientation at an earlier timepoint.

## 1. Introduction

Progression-free survival (PFS) is a clinically relevant and validated endpoint for the effect of first-line treatment in metastatic colorectal cancer (mCRC), independent of subsequent treatment lines. At present, the gold standard for response evaluation is RECIST 1.1 (Response Evaluation Criteria in Solid Tumors version 1.1) [1], which has limitations regarding interobserver reproducibility and non-measurable disease [2]. Moreover, a substantial increase in tumor size or new lesions are required for objective progression. A universal biomarker might be a parameter for an early and more reliable evaluation of the treatment effect than RECIST 1.1.

Circulating tumor specific DNA (ctDNA) represents an attractive biomarker in plasma that can be measured as a fraction of circulating total cell free DNA (cfDNA) and has potential for real-time monitoring of tumor status in patients with solid tumors [3]. It may be used as a liquid biopsy overcoming problems related to tumor heterogeneity; it is minimally invasive and easy to repeat [4]. Mutated ctDNA has been investigated in several studies of mCRC and seems to serve as an early marker of treatment effect [5,6]. This, however, requires knowledge of tumor specific mutations, and, in colorectal cancer (CRC) the most frequent mutations, RAS and RAF, are found in approximately 50–60% of tumors [7]. A universal biomarker present in the majority of patients with mCRC is crucial for improving precision medicine in this group of patients.

Epigenetic alterations, i.e., aberrant methylation of DNA, play an important role in carcinogenesis and represent another approach for measuring ctDNA. Changes in the methylation of the promoter region of a gene affect the transcription of the gene; hypomethylation usually increases gene transcription, while hypermethylation inhibits transcription [8]. The silencing of specific genes, such as tumor suppressor genes and DNA repair genes, affects growth arrest and cell-cycle control, with importance for tumor growth [9]. Aberrant methylation occurs in almost all malignant tumors, and this universal nature of methylation gives potential for methylated ctDNA as a more broadly applicable marker than mutated ctDNA, which is supported by recent publications measuring hypermethylated ctDNA (meth-ctDNA) in the plasma of most patients with mCRC [10,11]. An analysis of methylation patterns in CRC found the gene expression of the neuropeptide Y gene (*NPY*) to be downregulated when the promoter region of the gene was hypermethylated [12]. *NPY* encodes the neurotransmitter neuropeptide Y (NPY), which is involved in cell invasion and proliferation [13,14]. It has been shown that NPY inhibits the invasive potential of colon cancer cells in vitro [13]. Roperch et al. proposed a panel of three tumor-specific hypermethylated genes, including *NPY*, for CRC diagnosis [15], while Garrigou et al. investigated the same panel for follow-up in different stages of CRC [10]. Hypermethylation of the *NPY* promotor region (meth-NPY) has, thus, been suggested as a promising biomarker in CRC [10,15,16], but its clinical utility remains to be established.

Meth-ctDNA can be measured with high sensitivity by droplet digital polymerase chain reaction (ddPCR) allowing for the detection of very small fractions of meth-ctDNA [17,18].

Our group previously presented survival data from the randomized, double-blind, placebo-controlled phase II FOLFOXIRI-Toco trial investigating 5-FU, oxaliplatin, and irinotecan (FOLFOXIRI) combined with δ-tocotrienol or placebo as supportive treatment in first-line treatment of mCRC [19]. The aim of the present retrospective study was to investigate meth-NPY as an early biomarker of treatment effect in patients treated in the FOLFOXIRI-Toco trial.

## 2. Materials and Methods

The study was approved by The Regional Committee on Health Research Ethics for Southern Denmark (S-20150185) and the Danish Medicines Agency (2015122439), and it was prospectively registered with ClinicalTrials.gov (NCT02705300). Reporting of the study followed the Reporting Recommendations for Tumor Marker Prognostic Studies (REMARK) criteria [20]. Written and orally informed consent to translational research according to the Helsinki II Declaration was obtained from all patients.

### 2.1. Study Population

Between May 2016 and December 2018, 70 patients with unresectable mCRC were included in the FOLFOXIRI-Toco study for first-line treatment at the Danish Colorectal Cancer Center South, Vejle Hospital, Denmark. All patients were treated with FOLFOXIRI and were randomized to receiving δ-tocotrienol or placebo. The inclusion criteria were adenocarcinoma in the colon or rectum, age 18–75 years, ECOG performance status (PS) 0–1 (patients above 70 years were eligible if PS = 0), and evaluable but not necessarily measurable disease according to RECIST 1.1. Previous adjuvant chemotherapy for radical treatment of stage II or III colorectal cancer was allowed in patients proven disease-free for more than 6 months. Treatment was evaluated every 8 weeks by CT scan according to RECIST 1.1. Treatment was discontinued in the case of progressive disease (PD), unacceptable toxicity, patient request, or death, or as decided by the treating physician.

### 2.2. Blood Sampling

Plasma samples were collected at baseline, before every treatment cycle (every 2 weeks), and at every follow-up visit (every 3 months the first year, every 6 months the second and third years, and yearly the fourth and fifth years) or until progression. Firstly, 18 mL of blood was drawn in EDTA tubes for marker analysis. The samples were centrifuged at 2000× *g* for 10 min within 4 h of sampling and stored at −80 °C until analysis.

### 2.3. Methylation Analysis

Meth-NPY was defined as DNA with methylation of the NPY gene promoter [13]. Prior to methylation analysis, plasma was centrifuged again at 10,000× *g* for 10 min. DNA was purified from 4 mL of plasma using the QIAsymphony circulating DNA kit (Qiagen, Hilden, Germany), and cysteine-rich polycomb-like protein 1 (CPP1) DNA fragments were added as an exogenous internal control [21]. Then, 20 µL of DNA was bisulfite-converted in a 150 μL reaction with the EZ DNA Methylation Lightning kit, (Zymo research, Irvine, CA, USA) and eluted in 15 μL. As negative controls, water and a pool of genomic DNA from whole blood were included in each round of analyses. Universal Methylated Human DNA Standard (Zymo Research) and EpiTect control DNA (Qiagen, Hilden, Germany) were included as positive controls. The converted DNA was analyzed with a methylation-specific assay and a control assay (albumin) using the BioRad Droplet Digital PCR system QX200 (BioRad, Hercules, CA, USA). Albumin/NPY duplex analysis [8] was performed in two wells with 5 μL of converted DNA per well in 20 μL reactions. DdPCR supermix for probes (no deoxyuridine triphosphate), and NPY/ALB assays were applied. Droplets were generated on the QX200 automated droplet generator from BioRad. PCR was run on a Veriti PCR device. The samples were read on the QX200 droplet reader from BioRad. Data analysis was performed with QuantaSoft ver. 1.7.4 (BioRad). The limit of blank (LOB) was defined from the number of NPY droplets counted in control DNA samples from a test cohort of 50 healthy individuals and validated in an independent cohort of healthy donors [22]. The upper 95% confidence interval (CI) was two droplets per reaction based on the Poisson distribution. Samples were considered positive when the number of observed droplets was higher than the LOB value. Plasma analysis was performed blinded to patient outcomes.

### 2.4. Definition of Groups and Endpoints

At baseline, patients were divided into two subgroups: above and below the median of meth-NPY. After the first treatment cycle, patients were further divided into two subgroups according to meth-NPY response: responders and non-responders. Response rate according to meth-NPY was defined as the fraction of patients converting from a baseline detectable level to an undetectable level after the first treatment cycle. The undetectable level comprised zero values and those with the lower 95% CI including zero.

During treatment and follow-up, a value with no overlap between the 95% CI of the current and preceding measurements was considered a significant increase in meth-NPY.

Patients with response according to RECIST 1.1 included those with partial response (>30% reduction in target lesions) and complete response. For patients undergoing surgery, the best response on imaging prior to surgery was registered. Patients with non-evaluable or unknown response were not included in the response analysis (*N* = 5).

Lead time was defined as the median time from a significant increase in meth-NPY to progression according to RECIST 1.1.

### 2.5. Data Management

The clinical data were stored and managed using the Research Electronic Data Capture (REDCap) tool [23,24] hosted by Open Patient data Explorative Network (OPEN), Odense University Hospital, Region of Southern Denmark.

### 2.6. Statistical Analysis

Baseline characteristics were compared using Fisher’s exact test and the Wilcoxon Mann–Whitney test for binary and continuous variables, respectively.

Response rate based on meth-NPY after one treatment cycle was correlated with survival and response according to RECIST 1.1. Survival analyses were calculated from the day of the first treatment to the date of progression or death of any cause or censored at surgery or last hospital contact (PFS) and to the date of death (OS), respectively. Follow-up was censored at the time of data work-up (November 2021). Median follow-up was calculated using the inverse Kaplan–Meier method. Kaplan–Meier curves illustrating survival were compared with the log-rank test, and a Cox regression model was used to estimate hazard ratios (HRs).

Multivariate survival analysis was performed using the Cox regression model with the proportional hazard assumption tested. Together with age, gender, and randomization to either δ-tocotrienol or placebo, the parameters entered in the multivariate Cox regression were variables with *p* < 0.20 in the univariate Cox regression analysis.

All reported *p*-values were two-sided, and *p*-values <0.05 were considered statistically significant. Statistical analyses were performed using STATA 17.0 Stata Corp. College Station, TX, USA.

## 3. Results

### 3.1. Patient Characteristics

Data cutoff for updated survival analyses was performed on 1 November 2021. The median follow-up time was 48.5 months.

Sixty-eight of 70 patients had blood sampled at baseline, and 66 of 69 had blood sampled after the first treatment cycle. Sixty-five patients were evaluable for response by RECIST 1.1. During chemotherapy, 16 patients underwent surgery for metastases. Nine patients had partial response on the last scan before surgery, and seven had stable disease.

Seven (10.3%) and 19 (28.8%) patients had an undetectable level of meth-NPY at baseline and after the first treatment cycle, respectively. Six patients had an undetectable level of meth-NPY at both timepoints. Baseline characteristics according to meth-NPY at baseline are shown in Table 1. There were no significant differences in the characteristics between the two subgroups.

The median level of meth-NPY was 15.45% (range 0–97.5%) at baseline and 1.15% (range 0–52%) after the first treatment cycle.

### 3.2. Prognostic Value of Meth-NPY at Baseline

**Figure 1:**
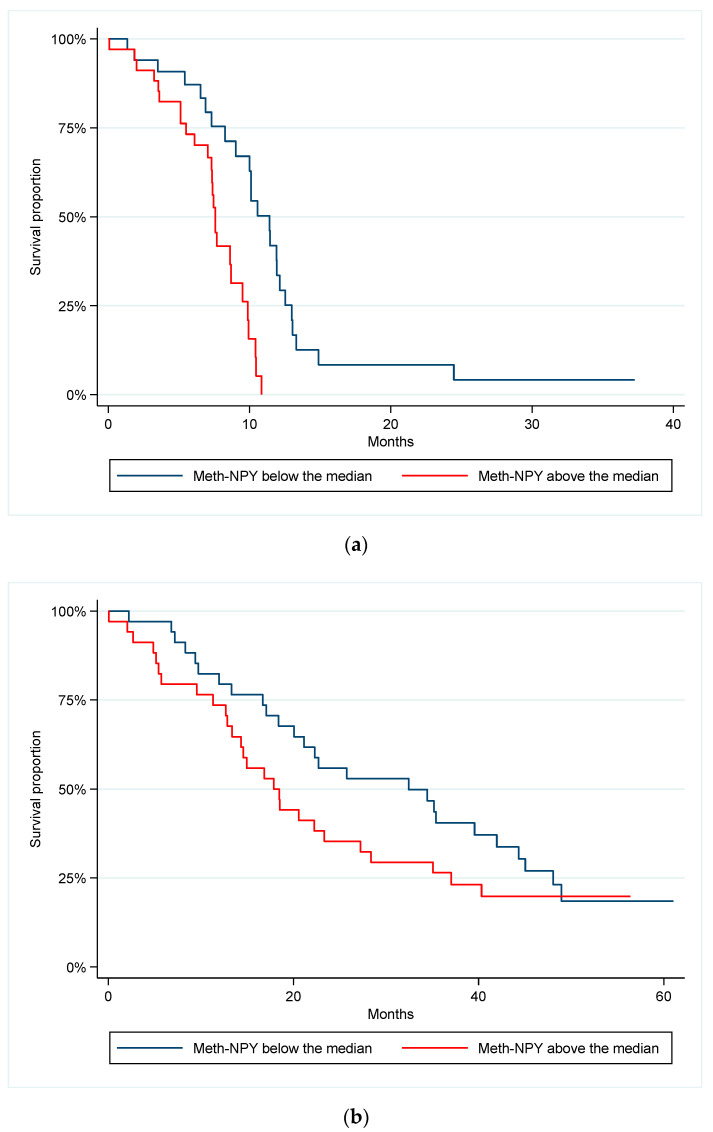
Kaplan–Meier plots describing (**a**) progression-free survival according to baseline level of meth-NPY above or below the median, *p* < 0.001, HR = 3.63 and (**b**) overall survival according to baseline level of meth-NPY above or below the median, *p* = 0.21, HR = 1.41.

The seven patients with undetectable meth-NPY at baseline had a median PFS of 11.9 months (95% CI 7.3–not reached (NR)) compared to 8.6 months in the 61 patients with detectable meth-NPY (95% CI 7.4–10.0) (log-rank *p* = 0.05, HR 2.3, 95% CI 0.97–5.65). Patients with undetectable and detectable meth-NPY at baseline had a median OS of 34.5 months (95% CI 16.7–NR) and 20.6 months (95% CI 15.0–32.5), respectively (log-rank *p* = 0.31, HR 1.61, 95% CI 0.64–4.06). Comparing patients with meth-NPY below and above the median at baseline resulted in a significant difference in PFS, 11.4 vs. 7.6 months (*p* < 0.001, HR 3.63, 95% CI 1.78–7.41). There was no significant difference in OS (32.5 vs. 17.9 months, *p* = 0.21, HR 1.41, 95% CI 0.82–2.43) (Figure 1a,b).

### 3.3. Early Treatment Effect

**Figure 2:**
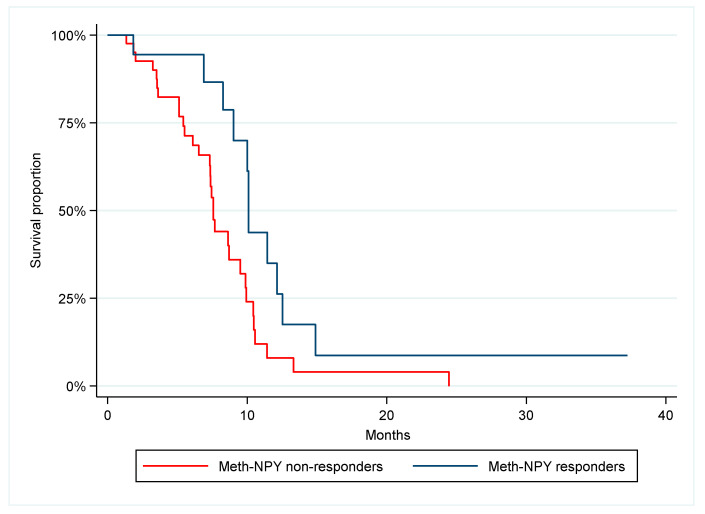
Progression-free survival according to meth-NPY response rate after the first treatment cycle. Median PFS was 10.1 vs. 7.6 months, *p* = 0.02, HR = 0.43.

**Figure 3:**
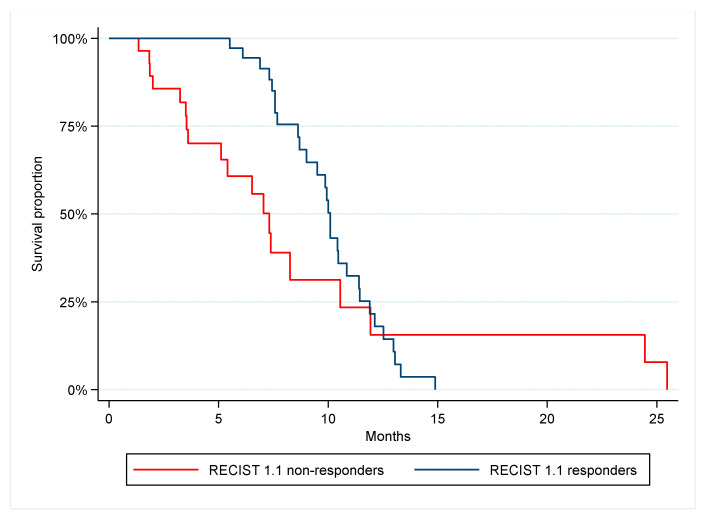
Progression-free survival according to RECIST 1.1 response. Median PFS was 10.1 vs. 7.3 months, *p* = 0.17, HR = 0.65.

Overall, the PFS was 9.0 months, and the OS was 22.3 months. Fifty-nine patients were evaluable for analysis of meth-NPY response after the first cycle and 65 patients were evaluable according to RECIST 1.1. The response rates according to meth-NPY and RECIST 1.1 were 31% and 57%, respectively. PFS was significantly longer in meth-NPY responders compared to non-responders, 10.1 and 7.6 months, respectively (*p* = 0.02, HR 0.43, 95% CI 0.21–0.88), (Figure 2). Patients with response according to RECIST 1.1 had a PFS of 10.1 months compared to 7.3 months for non-responders, which was not statistically significant different (*p* = 0.17, HR 0.65, 95% CI 0.35–1.21), (Figure 3). No statistically significant difference was found in OS between meth-NPY response (*p* = 0.15) and RECIST 1.1 (*p* = 0.15) (Figures S1 and S2).

In addition to meth-NPY response after the first cycle, PS was correlated with PFS in the univariate analysis and included in the multivariate analysis together with age, gender, and randomization to δ-tocotrienol or placebo. In the multivariate analysis, meth-NPY response (HR 0.35, *p* = 0.008) and PS (HR = 2.1, *p* = 0.02) remained prognostic factors (Table 2).

### 3.4. Meth-NPY Lead Time

At the time of data cutoff, five patients were still without progression. During chemotherapy treatment and follow-up, respectively, 28 (40%) and 34 (49%) patients progressed. One patient (1%) died during the first treatment cycle, and two patients (3%) transferred their follow-up to another hospital and were censored before progression.

Ten patients had undetectable meth-NPY at progression. The blood sample at progression was missed in one patient. Calculation of lead time was performed in 51 patients with a median of 49 days (range 0–155). Patients progressing during chemotherapy (including the period from last treatment to response evaluation after 4 weeks) had a median lead time of 49 days (range 0–119) compared to 51 days (range 0–155) in patients progressing in the follow-up period.

## 4. Discussion

Early meth-NPY response has potential as a minimally invasive tool to predict treatment effect, and, in this study of first-line treatment in mCRC, early meth-NPY response performed better in predicting PFS than response according to RECIST 1.1.

The latest RECIST guideline (revised RECIST 1.1) was published in 2008, and, despite several limitations, it is still the gold standard of evaluation in mCRC [1]. In a literature-based analysis of 50 first-line trials on mCRC, there was a high correlation between PFS and OS in chemotherapy regimens, justifying PFS as a surrogate endpoint for OS [25]. PFS is currently used in mCRC trials as the primary endpoint; it is independent of subsequent treatments and was the focus of survival in the present study.

Several studies are pointing toward ctDNA as a reliable tool in evaluation of treatment effect in mCRC. Our group previously investigated meth-NPY in 123 patients with mCRC receiving first line treatment with 5-FU, oxaliplatin, and bevacizumab, divided into two subgroups [26]. Of note, the subgroup with the longer survival included patients with response according to meth-NPY, as well as those with undetectable meth-NPY at baseline and after the first treatment cycle. The latter were not included in the present study. An early marker of treatment effect was also investigated in the PLACOL study by Garlan et al., where mutated (KRAS, BRAF, or TP53) and methylated ctDNA (*WIF1* and *NPY*) were analyzed in 73 patients [27]. In evaluating the predictive impact of ctDNA between baseline and before the third treatment cycle, they found a significant difference in PFS and OS when ctDNA decreased to a negligible level below 0.1 ng/mL. Their cohort differed from ours in including both first- and second-line treatment and targeted treatment. These studies elucidate a potential correlation between initial dynamics in meth-NPY and early prediction of treatment effect, although, in our study, the difference in OS was not statistically significant.

Meth-NPY response challenges the RECIST guideline as to evaluation of patients treated with systemic therapy. In our study, an early NPY response 2 weeks after the first treatment was superior to RECIST 1.1. in predicting PFS. The meth-NPY response rate after one treatment cycle was 31% compared to the objective response rate of 57%, but the higher objective response rate did not reflect a significant difference in PFS. This places meth-NPY as a promising early marker compared to imaging where best response is often achieved up to several months after treatment start. Early meth-NPY response gives an opportunity for drug evaluation in trials earlier than today’s standards. In the multivariate analysis, meth-NPY response was the strongest variable, and randomization between δ-tocotrienol or placebo was not prognostic.

The hypothesis of discovering treatment resistance earlier in the blood than by imaging was proven by a significant increase in meth-NPY in plasma with a median of 49 days before progression was found on the conventional CT scan. Other studies from our center support this finding in which mCRC patients receiving standard first-line treatment [28] or last-line regorafenib [29] had a median lead time to progression of 51 days (range 14–133 days) and 50 days (range 14–255 days), respectively.

In many patients, chemotherapy resistance is visible in the blood earlier than on imaging. Some patients are, therefore, potentially exposed to ineffective, harmful treatment, and their access to other potentially effective treatments is delayed. This suggests serial assessment of meth-ctDNA as a supplement to imaging to improve monitoring in mCRC by applying it between radiological assessments as suggested by Barault et al. [11]. The group investigated meth-ctDNA as a tool for monitoring treatment response in 45 mCRC patients using a 5-methylation marker assay, but it is worth noting that only eight patients received conventional chemotherapy.

Using immune checkpoint inhibitors in the treatment of a subgroup of patients with mCRC has added another challenge to response evaluation by imaging due to pseudo-progression [30,31], but the value of meth-NPY remains to be investigated in that setting.

The reporting on ctDNA monitoring of treatment effect in metastatic cancer has recently been discussed [32]. Currently, the comparison of relevant studies on ctDNA is hampered by the diversity in reporting. In addition to lacking definitions of the optimal timepoints for repeated measurement, this complicates the translation into clinical utility.

Mutated ctDNA is broadly investigated in mCRC and holds promise as an early marker of treatment effect in many patients, but the most common mutations are only found in about half of mCRC patients, leaving a subgroup in which another type of biomarker is required. Searching for random mutations by whole-genome sequencing or next-generation sequencing in these patients is a time-consuming and expensive procedure, and it is not available in every laboratory. Meth-ctDNA can be detected by ddPCR, a fast and less expensive procedure.

In relation to applying meth-NPY as a universal biomarker in mCRC, attention must be paid to the fact that, in 10.3% of the patients in our study, meth-NPY was undetectable at baseline. Other studies found the marker to be undetectable in 14–20% of their populations at baseline [11,26]. Barault et al. reported individual markers to have a prevalence of around 65% and achieved a prevalence of 86% by combining the positivity of 5-methylated loci [11]. Addition of other meth-ctDNA markers to meth-NPY might increase the detectability and warrants further studies. We suggest randomized marker-driven clinical trials with a monitoring/follow-up design comparable to the OPTIMISE trial (http://clinicaltrials.gov/ct2/show/NCT04680260 (accessed on 25 October 2021)).

The strengths of the study include the prospective design providing reliable data quality and the high internal validity offered by a single-center study. ctDNA was analyzed batch-wise to reduce variation, and the universal characteristic of methylated ctDNA reduces the need for several mutation analyses. Our study is limited by the relatively small number of patients, the retrospective analysis, and the fact that there is no validation cohort, which impairs the generalization of our conclusions. Furthermore, the potential biological variation in measurements was not taken into account [33].

## 5. Conclusions

Meth-NPY as an early measure of response to first-line treatment of mCRC proved to be superior to RECIST 1.1 with respect to predicting PFS. Meth-NPY response is an early indicator of clinical benefit and a promising endpoint in clinical trials. A significant increase in meth-NPY during treatment or follow-up is an early indicator of progression allowing treatment reorientation at an earlier timepoint.

## Figures and Tables

**Table 1:** Baseline characteristics.

Variabel	Total *N* = 70	Baseline Sample *N* = 68	Undetectable Meth-NPY *N* = 7	Detectable Meth-NPY *N* = 61	*p*-Value
Age, median (range)	64 (40–75)	64 (40–75%)	59 (54–67%)	64 (40–75)	0.12
Gender					0.70
Female	27 (39%)	26 (38%)	2 (29%)	24 (39%)	
Male	43 (61%)	42 (62%)	5 (71%)	37 (61%)	
PS					1.00
0	48 (69%)	46 (68%)	5 (71%)	41 (67%)	
1	22 (31%)	22 (32%)	2 (29%)	20 (33%)	
Tumor location					0.91
Right colon	21 (30%)	21 (31%)	2 (29%)	19 (31%)	
Left colon	29 (41%)	28 (41%)	3 (43%)	25 (41%)	
Rectum	20 (29%)	19 (28%)	2 (28%)	17 (28%)	
Primary metastatic disease	47 (67%)	46 (68%)	3 (43%)	43 (70%)	0.20
Recurrent disease	23 (33%)	22 (32%)	4 (57%)	18 (30%)	
Previous adjuvant chemo	6 (9%)	6 (9%)	2 (29%)	4 (7%)	0.11
RAS/RAF mutation	49 (70%)	48 (71%)	5 (71%)	43 (70%)	1.00
Liver-only disease	16 (23%)	16 (24%)	1 (14%)	15 (25%)	1.00

*N*, number; PS, performance status.

**Table 2:** Multivariate analysis.

Variabel	PFS	
	HR (95% CI)	*p*-Value
Meth-NPY		0.008
Non-responders	Reference	
Responders	0.34 (0.16–0.76)	
Age	0.98 (0.94–1.02)	0.46
Gender		0.39
Male	Reference	
Female	1.34 (0.69–2.6)	
PS		0.024
0	Reference	
1	2.10 (1.1–4.0)	
Randomization		0.34
Placebo	Reference	
Tocotrienol	1.40 (0.71–2.77)	

PFS, progression-free survival; HR, hazard ratio; CI, confidence interval.

## Data Availability

Patient data in this study are available on request to the corresponding author. The data are not publicly available due to privacy (General Data Protection Regulation) and ethical reasons.

## Supplementary Materials

The following supporting information can be downloaded at https://www.mdpi.com/article/10.3390/cancers14184459/s1: Figure S1. Overall survival according to meth-NPY response after the first treatment cycle title; Figure S2. Overall survival according to RECIST 1.1 response.
