# Supplementary material for: NPY Gene Methylation in Circulating Tumor DNA as an Early Biomarker for Treatment Effect in Metastatic Colorectal Cancer
Source: Cancers (Basel). 2022 Sep 14;14(18):4459. doi: 10.3390/cancers14184459 (PMC9496936; doi:10.3390/cancers14184459)
Supplement: Supplementary file 1 [file cancers-14-04459-s001.zip › cancers-1841048-supplementary.pdf]

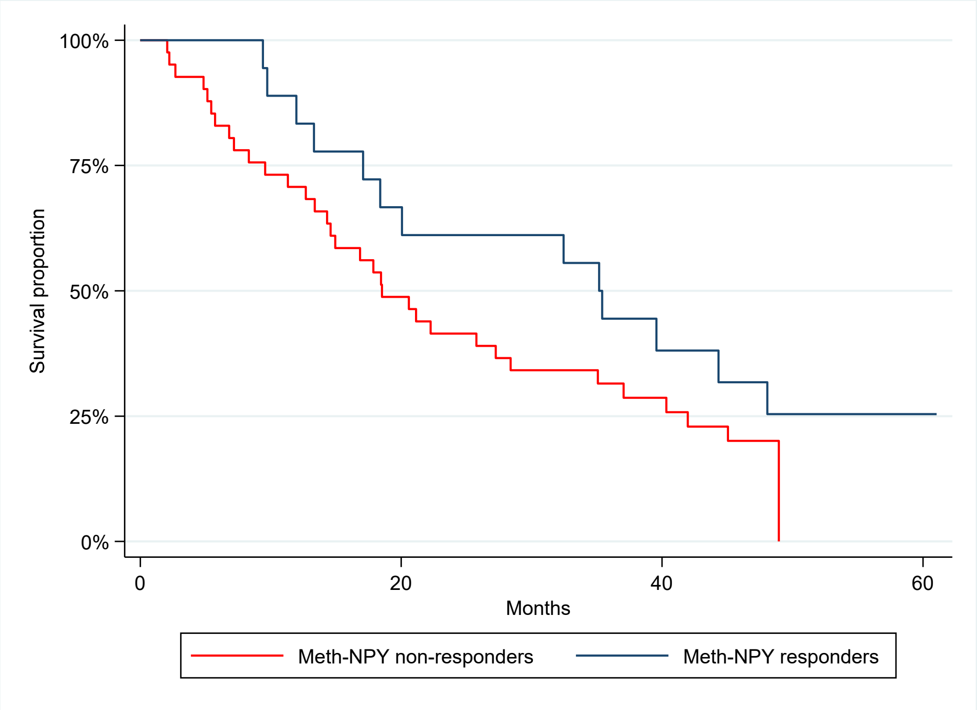

Figure S1: Overall survival (OS) according to meth-NPY response after the first treatment cycle. Median OS was 35.2 vs 18.5 months in responders vs non-responders, respectively ($p = 0.15$, HR = 0.62)

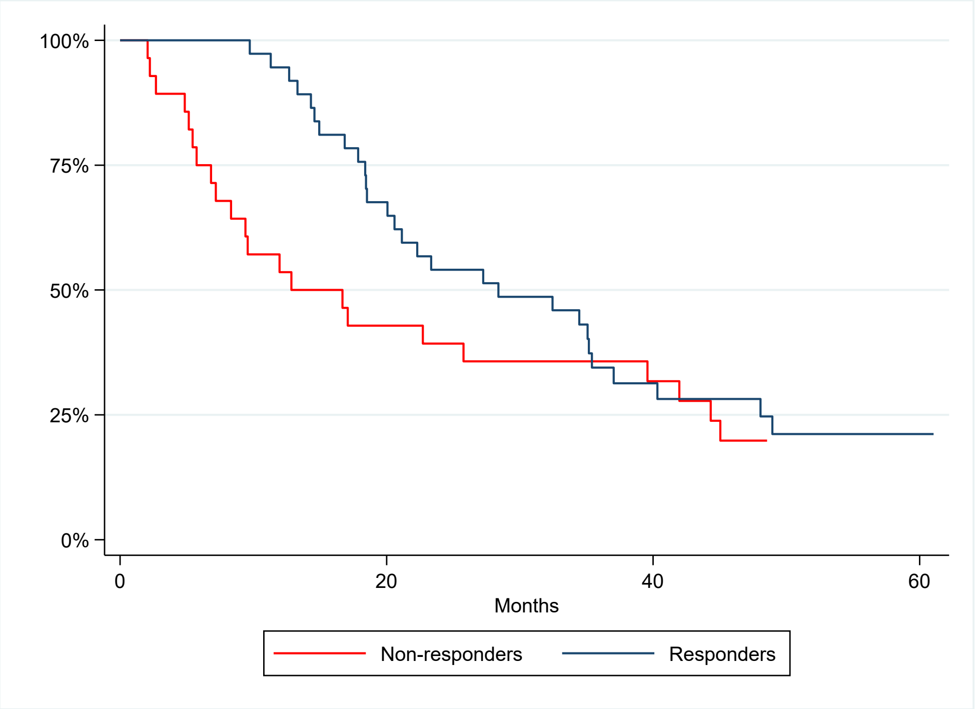

Figure S2: Overall survival (OS) according to RECIST 1.1 response. Median OS was 28.4 vs. 12.9 months in responders vs non-responders, respectively ($p = 0.15$, HR = 0.66)
